# *De Novo* Assembly of *Auricularia polytricha* Transcriptome Using Illumina Sequencing for Gene Discovery and SSR Marker Identification

**DOI:** 10.1371/journal.pone.0091740

**Published:** 2014-03-13

**Authors:** Yan Zhou, Lianfu Chen, Xiuzhi Fan, Yinbing Bian

**Affiliations:** 1 Institute of Applied Mycology, Huazhong Agricultural University, Wuhan, Hubei, China; 2 Key Laboratory of Agro-Microbial Resource Comprehensive Utilization, Ministry of Agriculture, Huazhong Agricultural University, Wuhan, Hubei, China; Oregon State University, United States of America

## Abstract

*Auricularia polytricha* (Mont.) Sacc., a type of edible black-brown mushroom with a gelatinous and modality-specific fruiting body, is in high demand in Asia due to its nutritional and medicinal properties. Illumina Solexa sequenceing technology was used to generate very large transcript sequences from the mycelium and the mature fruiting body of *A. polytricha* for gene discovery and molecular marker development. *De novo* assembly generated 36,483 ESTs with an N50 length of 636 bp. A total of 28,108 ESTs demonstrated significant hits with known proteins in the nr database, and 94.03% of the annotated ESTs showed the greatest similarity to *A. delicata*, a related species of *A. polytricha*. Functional categorization of the Gene Ontology (GO), Clusters of Orthologous Groups (COG) and Kyoto Encyclopedia of Genes and Genomes (KEGG) metabolic pathways revealed the conservation of genes involved in various biological processes in *A. polytricha*. Gene expression profile analysis indicated that a total of 2,057 ESTs were differentially expressed, including 1,020 ESTs that were up-regulated in the mycelium and 1,037 up-regulated in the fruiting body. Functional enrichment showed that the ESTs associated with biosynthesis, metabolism and assembly of proteins were more active in fruiting body development. The expression patterns of homologous transcription factors indicated that the molecular mechanisms of fruiting body formation and development were not exactly the same as for other agarics. Interestingly, an EST encoding tyrosinase was significantly up-regulated in the fruiting body, indicating that melanins accumulated during the processes of the formation of the black-brown color of the fruiting body in *A. polytricha* development. In addition, a total of 1,715 potential SSRs were detected in this transcriptome. The transcriptome analysis of *A. polytricha* provides valuable sequence resources and numerous molecular markers to facilitate further functional genomics studies and genetic researches on this fungus.

## Introduction

*Auricularia polytricha* (Mont.) Sacc., a type of edible black-brown mushroom rich in various nutrients such as polysaccharides, edible fibers and proteins, is widely distributed in Asia, tropical America, and other regions around the world. *A. polytricha* has been a common culinary ingredient in East Asia for hundreds years. This species is in high demand in Asia due to its nutritional and medicinal properties [1].

*A. polytricha* is classified within the Basidiomycota, Agaricomycetes, Incertae sedis, Auriculariales, Auriculariaceae, *Auricularia* [2]. The fruiting bodies of *A. polytricha* lacks the obvious tissue differentiation of pileus, gill and stipe, which is distinct from other Agaricomycetes fungi such as *Schizophyllum commune*, *Coprinopsis cinerea*, *Agaricus bisporus* and *Lentinula edodes*, and the fruiting bodies are able to survive for a long period of drought. Despite the unique appearance in the fruiting body of *A. polytricha*, it is developed from filamentous mycelium like other mushrooms. Unfortunately, insufficient genomic information is available for *A. polytricha*, which has limited our understanding of the molecular mechanism underlying the formation and development of its special fruiting body.

In recent years, next-generation sequencing techniques (such as Roche 454, Illumina Solexa GA and ABI SOLID) have emerged as a cutting-edge approach for high-throughput sequence determination, providing powerful and cost-efficient tools for advanced research in many areas, including genome sequencing; genome-wide profiling of epigenetic marks and chromatin structure using ChIP-seq, methyl-seq and DNase-seq; metagenomics studies; and *de novo* transcriptome sequencing for non-model organisms [3], [4]. Transcriptomic information is used in a wide range of biological studies and provides fundamental insight into biological processes and applications, such as profiles of the gene expression levels during different space-time conditions, gene discovery and molecular marker mining [5], [6], [7]. Several studies have reported the genome or transcriptome sequencing of various mushrooms, including *S. commune* [8], *C. cinerea* [9], *L. edodes* [10], [11], *Ganoderma lucidum* [12], [13], *Agrocybe aegerita* [14] and *Cordyceps militaris* [15], [16]. Fruiting body formation is one of the most complex developmental processes in mushrooms [17]. Transcriptomes and comparative expression profiles from different developmental stages have been generated to explore many genes associated with fruiting body formation. A set of transcription factors (TFs) acting downstream of the mating-type loci has been identified in the model mushroom *S. commune*, and a model for the regulation of mushroom development has been constructed based on the phenotype variations that occur with different mutations of TFs [8], [18].

Due to the deficiency of information on the complex molecular mechanisms of *A. polytricha* development, the objective of the current study was to use the Illumina sequencing technology to determine the transcriptomes of mycelium and fruiting body of *A. polytricha*. A total of 36,483 ESTs were obtained by *de novo* assembly, and an analysis of the differentially expressed genes (DEGs) revealed candidate genes involved in the fruiting body formation of *A. polytricha*, including homologs of TFs which play important roles in the fruiting body development of other mushrooms. The assembled, annotated transcriptome dataset and gene expression profiles provide an invaluable genomic resource for future research in *A. polytricha*. Furthermore, the EST-based SSR markers identified in this study can be applied to analyze the genetic diversity and to construct a transcript map of *A. polytricha*, which will be essential for accelerating mushroom breeding programs in the future.

## Materials and Methods

### Mushroom Strains and Cultivation

The dikaryotic *A. polytricha* strain APM2-16 is a hybrid of two monokaryotic strains, App7 and M2S16. App7 was isolated from the wild dikaryotic strain APTJ6101 by protoplast regeneration, and M2S16 was derived from the cultivated strain MHJY002 by single-spore isolation.

The mycelia of App7, M2S16 and APM2-16 were grown on liquid complete yeast medium (CYM) separately for one week at room temperature [19]. APM2-16 was cultivated on packs of growth medium consisting of 80% sawdust, 19% wheat bran and 1% CaSO_4_ in the dark at 25°C. When the packs were fully covered with mycelia, the culture surfaces were wounded using a knife, and all of the packs were transferred to conditions that induced fruiting (light, 15–25°C, >85% humidity). Some tissues like brains, consisted with many 1–3 mm primordia, grown on the wounded position after 5–7 d. The next two or three days, the primordia grown up to 3–5 mm young fruiting bodies, which resembled small cups. Then the following 7–15 d, the cup-like fruiting bodies were growing up and expanding to form ear-like fruiting bodies, which were more than 100 mm in longitudinal diameter and containing many mature basidiospores. The samples containing liquid cultured mycelia, primordia, young fruiting bodies and mature fruiting bodies were immediately frozen in liquid nitrogen and stored at −80°C until RNA extraction.

### cDNA Library Preparation and Illumina Sequencing

Total RNA from dikaryotic mycelium and adult fruiting body of APM2-16 was extracted using TRIzol Reagent (Invitrogen Life Technologies, USA) according to the manufacturer’s protocol. The integrity of the RNA was detected by agarose gel electrophoresis and the concentration was determined using a NanoDrop 2000 spectrophotometer (Thermo Scientific, Wilmington, DE, USA). The RNA quality of the dikaryotic mycelia and adult fruiting bodies for RNA-seq was further verified using an Agilent 2100 Bioanalyzer (Agilent Technologies, Santa Clara, CA, USA). A total of 20 μg RNA was equally isolated from mycelia and adult fruiting bodies for mRNA isolation, cDNA library construction and sequencing according to the manufacturer’s instructions (Illumina, San Diego, CA, USA). The two cDNA libraries were sequenced at Beijing Genomics Institute (BGI)-Shenzhen, Shenzhen, China. The raw Illumina sequencing data for the mycelium (accession number: SRX319468) and adult fruiting body (accession number: SRX319472) generated in this study were submitted to the NCBI Sequence Read Archive database (SRA).

### *De novo* Assembly of Sequencing Reads

Before assembly, the 90-bp raw paired-end reads were filtered to obtain the high-quality clean reads by removing adaptors, low-quality sequences (reads with unknown bases “N”) and reads with more than 20% low quality bases (quality value ≤10). The clean reads from mycelium and fruiting body were mixed together and *de novo* assembly of the clean reads was performed using short reads assembling program Trinity [20]. Briefly, clean reads with a certain overlap length were combined to form longer fragments without N (contigs). The individual reads were assigned to the respective contigs by paired-end mapping. Trinity was able to detect contigs from the same transcript and determine the distances between these contigs. Finally, Trinity connected these contigs into sequences that could not be extended on either end, which were called transcripts. When multiple samples from the same species were sequenced, the transcripts assembly should be taken into further process of sequences splicing and redundancy removing. After clustering with BLASTx, the transcripts were divided into two classes: clusters (similarity >70%) and singletons. The clusters were the transcripts produced by alternative splicing or homologous genes. The ESTs containing the longest sequence of every cluster and all of the singletons were used for further bioinformatic analysis.

### Gene Annotation and Analysis

The sequence orientations of the ESTs were determined by BLASTx alignment (*E* value <10^−5^) against the NCBI non-redundant protein (nr) database, the Swiss-Prot protein database, the Kyoto Encyclopedia of Genes and Genomes (KEGG) pathway database and the Cluster of Orthologous Groups database (COG). If the results of different databases are in conflict, a priority of nr, Swiss-Prot, KEGG and then COG was followed when deciding the sequence direction of ESTs. When an EST did not align to the above databases, ESTScan was used to predict the sequence direction [21].

For functional annotation, EST sequences were aligned by BLASTx to the above protein databases (*E* value <10^−5^). The BLAST results of the best hit were extracted for EST description. Based on the results of nr annotation, the Blast2GO program was employed to obtain GO annotation according to molecular function, biological process and cellular component [22]. The EST sequences were also aligned to the COG database to predict and classify their possible functions [23]. The KEGG database was used to study the metabolic pathways in *A. polytricha* [24].

For the identification of TF families represented in the *A. Polythricha* transcriptome, the ESTs were searched against all the TF protein sequences in the Fungal Transcription Factor Database (FTFD, http://ftfd.snu.ac.kr) using BLASTx with an *E*-value cut-off of <10^−5^ [25]. To identify TF families, the protein sequences of all ESTs were functionally annotated using InterProScan [26].

### Analysis and Experimental Verification of Differentially Expressed Genes

The clean reads from dikaryotic mycelium and mature fruiting body were mapped on the ESTs respectively. If less than 10 reads from this two different cDNA libraries were mapped back on to one EST, this EST was considered to be expressed in low abudance and did not analyze the diversity of expression level among two developmental stages further. The RPKM (reads per kilobase per million reads) was applied to measure the gene expression levels [27]. The calculated gene expression was used to compare the differences in gene expression between mycelium and fruiting body. The probability of an EST being expressed equally between the two samples was calculated, and the false discovery rate (FDR) method was introduced to determine the threshold *p*-value in multiple tests. An FDR ≤0.001 and an absolute value of the log2 ratio ≥2 were used as thresholds to determine the significance of gene expression differences between the two developmental stages [28]. For functional enrichment analysis, the *p*-values for a significant overrepresentation of a particular GO category were calculated using the hypergeometric distribution. Pathway enrichment analysis was performed similarly using the KEGG database.

To assess the reliability of the sequencing-based approach in identifying differentially expressed genes, semi-quantitative RT-PCR was performed to detect gene expression levels using specific primers. Approximately 2 μg of total RNA from mycelium and fruiting body, treated with DNaseI (Fermentas), was used as a template to synthesize first-strand cDNA by reverse transcriptase M-MLV (TaKaRa). The gene-specific primers (Table S1) were designed based on the concerned EST sequences. Actin (comp28675_c0) and GAPDH (comp26162_c0) primers were used to standardize RNA samples for each RT-PCR. The expression of each gene was confirmed by performing independent PCR reaction three times.

### Gene Expression Profiles of TFs in Different Developmental Stages

Some TF genes have been determined their important roles in other mushrooms. The homologous TF genes in *A. delicata* genome was found using BLASTp. According to the functional annotation result of *A. polytricha* transcriptome, the ESTs with top hit against the corresponding protein of *A. delicata* were considered the homologous TF genes. The ESTs found in the same BLAST results or with high homology to one another were selected as allelic variants of different parts or alternative spliced transcripts of the same gene. In order to determine the function of homologous TF genes of *A. polytricha* in the development of fruiting body, the gene expression profile of these genes were estimated using semi-quantitative RT-PCR. Total RNA was isolated from the liquid-cultured mycelia of App7, M2S16 and APM2-16, as well as the primordia, young fruiting bodies and mature fruiting bodies of APM2-16. About 2 μg of total RNA of six different developmental stages was reverse-transcribed by reverse transcriptase M-MLV (TaKaRa). The gene-specific primers (Table S1) were designed based on one of the EST sequences of homologous TFs. *Actin* (comp28675_c0) and *GAPDH* (comp26162_c0) primers were amplified as endogenous loading control for testing the validity of template preparation. The expression of each gene was confirmed in at least three rounds of independent RT-PCR reactions.

### *In silico* SSR Identification

The software SSR Locator was employed to identify mono- to hexa-nucleotide SSR motifs [29]. Mono-nucleotide repeats of more than 15, di-nucleotide repeats of more than 8, tri-nucleotide repeats of more than 5, tetra-nucleotide repeats of more than 4 and penta- and hexa-nucleotide repeats of more than 3 were utilized as search criteria for SSRs.

## Results and Discussion

### Sequencing of *A. polytricha* Transcriptome and De Novo Assembly

For the purpose of generating an overview of the *A. polytricha* gene expression profiles of the different developmental stages, RNA was extracted from *A. polytricha* dikaryotic mycelia and mature fruiting bodies. High-throughput sequencing was performed with the Illumina HiSeq 2000 sequencing platform. After quality filtering, 10,130,920 and 10,434,094 clean paired-end sequence reads were obtained from the mycelium and fruiting body, respectively, with the Q20 percentage over 98% (Table 1). Then, using Trinity software, a total of 20,565,014 high-quality reads (consisting of 1,804,546,499 bp) were assembled into 524,847 contigs. The contigs were further assembled into 45,830 transcripts. After clustering with BLASTx, 36,483 ESTs were assembled from the *A. polytricha* transcriptome, including 5,022 distinct clusters and 31,461 singletons (Table 2). The mean length of ESTs was 531 bp and the N50 length was 636 bp (Table 2). The contig size distribution revealed that more than half of the ESTs (32,602; 89.4%) were between 200 and 1000 bp in length (Table 2). The GC content of *A. polytricha* ESTs was 61.31%, which was much higher than *L. edodes* (48.31%) and *A. aegerita* (approximately 53%) [11], [14]. GC content analysis revealed that *A. polytricha* transcripts have a high GC content similar to the *Auricularia delicata* genome (58.6%).

**Table 1:** Throughput and quality of Illumina sequencing of *A. polytricha* transcriptome.

Samples	Raw data		Clean data	
	Mycelium	Fruiting body	Mycelium	Fruiting body
Total reads	12,627,212	13,266,670	10,130,920	10,434,094
Total nucleotides (nt)	1,136,449,080	1,194,000,300	889,872,683	914,673,816
Q20 percentage^a^	90.88%	90.37%	98.42%	98.48%
N percentage^b^	0.04%	0.01%	0.04%	0.00%

a indicates the percentage of sequences at a sequencing error rate of less than 1%;

b indicates the percentage of the nucleotides that could not be sequenced.

**Table 2:** General features of the *A. polytricha* transcriptome.

	Transcripts	ESTs
Number of clean reads	20,565,014	
Total nucleotides (bp)	1,804,546,499	
Average read length (bp)	90	
GC content (%)	61.31	
Total number	45,830	36,483
Total length of sequences (bp)	25,781,703	19,384,088
Average length (bp)	562	531
N50 length (bp)	697	636
200–500 bp	28,022	23,606
500–1000 bp	12,020	8,996
1000–1500 bp	3,827	2,608
1500–2000 bp	1,382	873
>2000 bp	579	400

Without a reference genome for assembly, an approximation of transcriptome coverage was made. The genome size of *A. delicata*, an affinis species of *A. polytricha*, was 75 Mb. The genome sizes of sequenced species in Agaricomycetes were ranged from 25.04 Mb (*Piriformospora indica*) to 100.24 Mb (*Leucoagaricus gongylophorus*) (NCBI, http://www.ncbi.nlm.nih.gov). Based on the hypothesis that the genome size of *A. polytricha* was ranged from 25 Mb to 100 Mb, approximately 1.8 Gb of transcriptome sequence data likely represented 18 to 72-fold coverage of the *A. polytricha* genome. The *de novo* assembly of lower eukaryotic transcriptomes is straightforward and more than 30-fold coverage is required for the full lengths of most yeast transcripts, whereas the *de novo* assembly of higher eukaryotic transcriptomes is much more challenging because of the larger sizes of the datasets and the difficulties involved in identifying alternatively spliced variants [30], [31]. Furthermore, a reference-based assembly can reconstruct full-length transcripts with less than ten-fold coverage [32]. Therefore, higher coverage and genomic sequencing of *A. polytricha* should be conducted for a more complete assembly of the transcriptome. In spite of the limitation in coverage, a large set of *A. polytricha* gene fragments can provide adequate information for further gene investigation and identification of molecular markers.

### Functional Annotation and Characterization of *A. polytricha* Transcripts

A total of 28,108 (77.04%) ESTs exhibited a significant hit with known proteins in the nr database and matched 13,273 unique protein accessions. It is possible that the ESTs matched to the same protein accessions, represented different alleles or alternative splicing events for one gene in *A. polytricha*. This result indicated that the Illumina paired-end sequencing project generated more than 13,273 genes of *A. polytricha*. Homology analysis indicated that 94.04% of annotated ESTs showed the greatest similarity to the known proteins of *Aricularia*, including *A. delicata* (26,430), *A. auricula-judae* (2) and *A. polytricha* (2). Additionally, 854 (3.04%) annotated ESTs were best matched to sequences from other species of Agaricomycetes, such as *Gloeophyllum trabeum* (86), *Serpula lacrymans* (69), *Trametes versicolor* (49), *C. cinerea* (32), *S. commune* (41), *Laccaria bicolor* (31) and so on (Table S2). Despite the limited protein information of *A. polytricha* in nr database, the genomic sequencing results for *A. delicata* provided much useful information for transcriptome annotation in this study. Interestingly, the remaining annotated ESTs (720) showed significant hits with various species belonging to other groups of fungi, plants and animals. However, 9,095 (32.96%) ESTs did not show significant similarity with any other proteins in nr database, and may represent *A. polytricha*-specific genes.

### Functional Classification

**Figure 1:**
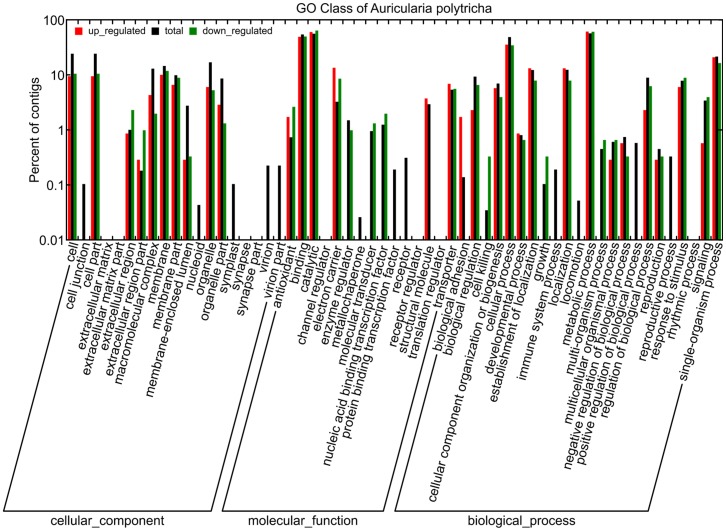
GO classification of the *A. polytricha* transcriptome and DEGs between the mycelium and fruiting body. The percentage of ESTs contained in a particular GO group is shown for all 11,513 ESTs (black), the 303 ESTs down-regulated (green) and the 347 ESTs up-regulated (red) in fruiting body.

GO is an international standardized gene functional classification system. Based on nr annotation, GO assignments were used to classify the functions of *A. polytricha* transcripts. Out of 28,108 nr hits, a total of 11,513 ESTs were assigned at least one GO term (Table S3). GO has three ontologies: cellular component, molecular function and biological process. WEGO software was applied to perform GO functional classification for the ESTs annotated with GO terms [33]. The 11,513 ESTs were categorized into 47 functional groups (Figure 1). Under the main category of cellular component, cell (2,811 ESTs, 24.42%) and cell part (2,811 ESTs, 24.42%) represented the majorities of the category. Binding activity (6,289 ESTs, 54.62%) and catalytic activity (6,491 ESTs, 56.38%) were dominant in the category of molecular function. Among the various biological processes, cellular process (5,635 ESTs, 48.94%) and metabolic process (6,585 ESTs, 57.20%) were most highly represented (Figure 1).

**Figure 2:**
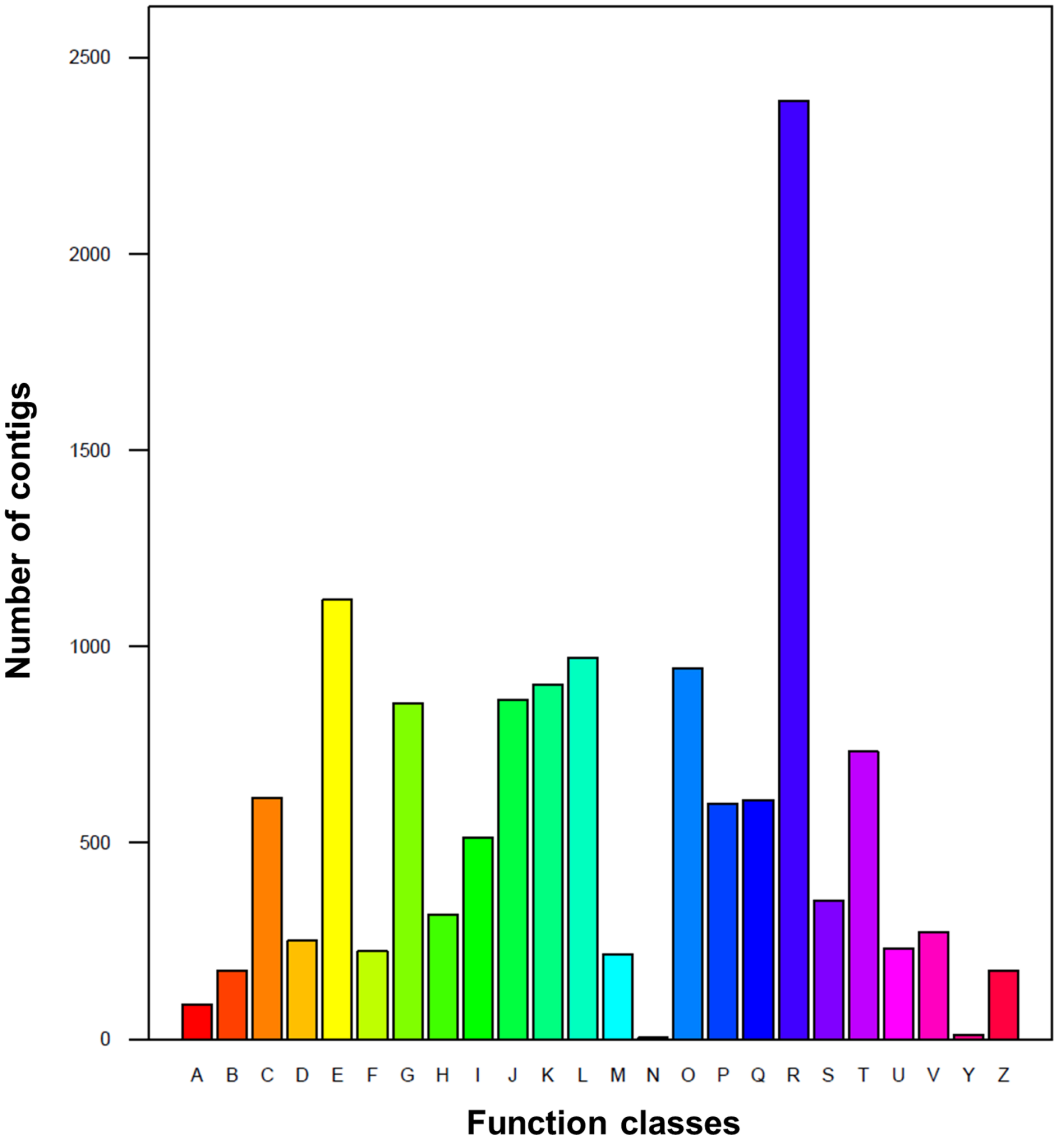
Histogram presentation of (COG) classification. A total of 9,705 *A. polytricha* ESTs were assigned to one or more COG functional categories. The letters on the x-axis represents different COG categories: RNA processing and modification (A), chromatin structure and dynamics (B), energy production and conversion (C), cell cycle control, cell division, chromosome partitioning (D), amino acid transport and metabolism (E), nucleotide transport and metabolism (F), carbohydrate transport and metabolism (G), coenzyme transport and metabolism (H), lipid transport and metabolism (I), transition, ribosomal structure and biogenesis (J), transcription (K), replication, recombination and repair (L), cell wall/membrane/envelope biogenesis (M), cell motility (N), posttranslational modification, protein turnover, chaperones (O), inorganic ion transport and metabolism (P), secondary metabolites biosynthesis, transport and catabolism (Q), general function prediction only (R), function unknown (S), signal transduction mechanisms (T), intracellular trafficking, secretion, and vesicular transport (U), defense mechanisms (V), nuclear structure (Y), cytoskeleton (Z).

In the COG database, every protein is assumed to be evolved from an ancestor protein. To further evaluate the completeness of the *A. polytricha* transcriptome and the effectiveness of the annotation process in this study, the annotated sequences were used to search for the genes involved in COG classifications [23]. A total of 9,705 ESTs were assigned to one or more COG functional categories. Among the 25 COG categories, “general function prediction only” was the largest group (1,805 ESTs, 18.60%), followed by “amino acid transport and metabolism” (842 ESTs, 8.68%). However, only a few ESTs were assigned to “cell motility” (4 ESTs, 0.04%) and “nuclear structure” (9 ESTs, 0.09%) (Figure 2). These results were different slightly to those obtained for *L. edodes*, *A. aegerita*, *C. cinerea*, *L. bicolor* and *S. commune* [11], [14].

To identify the biological pathways in *A. polytricha*, the 28,108 annotated sequences were mapped to the reference canonical pathways in the KEGG database. Pathway-based analysis helps further elucidate the biological functions and interactions of genes. A total of 2,676 ESTs had significant matches in the KEGG database and were assigned to 239 KEGG pathways (Table S4). A total of 1,172 ESTs were assigned to KEGG metabolism pathways. The pathways containing the most ESTs were involved in “amino acid metabolism” (265 ESTs), followed by “carbohydrate metabolism” (248 ESTs), “energy metabolism” (193 ESTs), “lipid metabolism” (174 ESTs) and “nucleotide metabolism” (166 ESTs), which are all involved in the maintenance of basic biological processes of *A. polytricha* (Table S4). These results showed that amino acid, carbohydrate and lipid metabolisms were active in this mushroom. In addition to the genes assigned to the metabolism pathways, 1,124 ESTs were classified into genetic information processing involving transcription, translation, folding, sorting, degradation, replication and repair, and 408 ESTs were sorted into cellular processes comprising cell communication, cell growth and death, cell motility, transport and catabolism. Moreover, a total of 216 ESTs were classified into membrane transport, signal transduction, signaling molecules and interaction. The KEGG annotations provided a valuable resource for investigating specific processes, functions and pathways involved in the *A. polytricha* vegetative growth of the mycelium and the development of the fruiting body.

**Figure 3:**
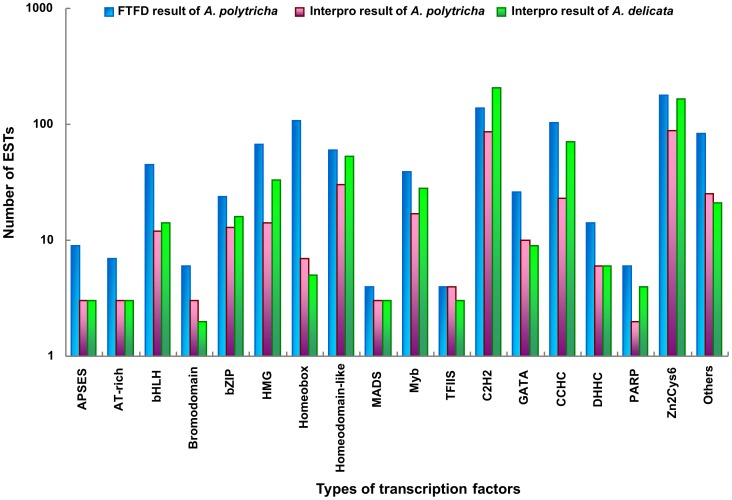
Distribution of *A. polytricha* and *A. delicata* putative TFs in different families. The “others” contained 16 different transcription factor families: Not1, centromere protein B, DDT, helix-turn-helix type 3, heteromeric CCAAT factors, negative transcriptional regulator, RFX DNA-binding domain, SART1, SGT1, ssDNA-binding, jumonji, WRKY, and GRF-type, MIZ-type, NF-X1-type, Rad18-type zinc finger.

Next, ESTs encoding putative TFs were identified by sequence comparison to the known TF gene families of fungi in the Fungal Transcription Factor Database [25]. In total, 1,349 putative TF-encoding ESTs, containing 924 ESTs similar to the sequences in FTFD, were identified, representing 3.70% of the *A. polytricha* transcriptome (Table S5, Table S6). A homology search for conserved protein domain was performed using InterProScan. Most ESTs were not complete ORF. Only 355 out of 1,349 putative TF-encoding ESTs were annotated in the InterPro database, distributed in 34 TF families (Table S6). 1,349 ESTs matched 502 unique protein accessions in nr database, suggesting that approximately 502 TFs might exist in the genome of *A. polytricha*, which was similar to the number of TFs in *S. commune* (Table S5) [8]. 23,577 protein of *A. delicata* genome released by the U.S. Department of Energy Joint Genome Institute (http://www.jgi.doe.gov) were also annotated using InterProScan. A total of 594 putative TFs were identified in the *A. delicata* genome. The ESTs of *A. polytricha* transcriptome containing the Zn2Cys6 fungal-specific zinc finger DNA binding domain were the most common TF family, followed by C2H2 zinc finger and CCHC-type zinc finger (Figure 3, Table S5). According to the InterPro results of *A. polytricha* transcriptome and *A. delicata* genome, the distribution of TFs among the various known protein families in *A. polytricha* and *A. delicata* were very similar (Figure 3), which differed somewhat from that of *S. commune* [8]. The *A* mating-type genes are potential homeobox TFs that contain a DNA-binding motif known as the homeodomain [34]. The *matA* locus of *S. commune* consists of two subloci: *Aα* and *Aβ*. The *Aα* locus contains two divergently transcribed genes, which encode the Y and Z homeodomain proteins of the HD2 and HD1 classes, respectively, while *Aβ* locus contains four genes encoding HD1 homeodomain proteins and two genes encoding HD2 [8]. Almost all of the eight *A* mating-type genes had low expression levels in the mycelium and fruiting body of *S. Commune* [8]. Although no reference genome of *A. polytricha* could be used to analyze the mating-type loci, some homologous *A* mating type ESTs were identified from the *A. polytricha* transcriptome for further investigations.

### Analysis of Differentially Expressed Genes

**Figure 4:**
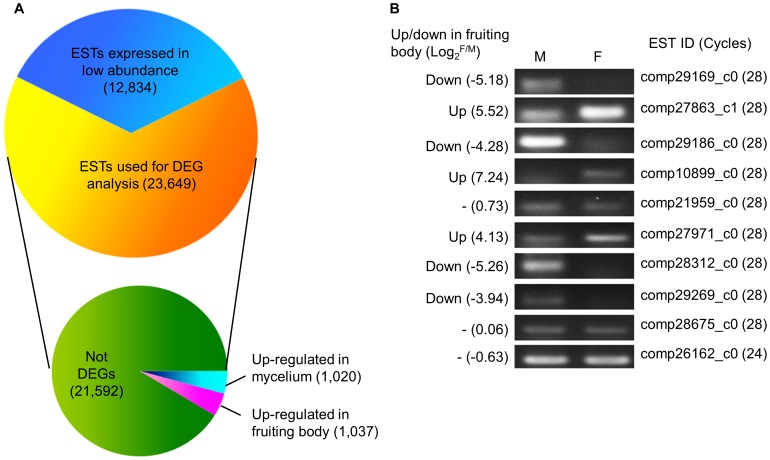
Overview and validation of DEGs between the mycelium and adult fruiting body of *A. polytricha*. (A) Out of 36,483 ESTs, expression levels of 12,834 ESTs at two developmental stages were too low to statistic analyze DEGs further. The remaining 23,649 ESTs were used for differential expression analysis. 2,057 ESTs were significantly differentially expressed between the mycelium and fruiting body (FDR ≤0.001, |log2-ratio| ≥2), including 1,020 ESTs down-regulated and 1,037 up-regulated in the fruiting body. (B) Validation of gene expression by semi-quantitative RT-PCR. M: dikaryotic mycelium; F: adult fruiting body. The captions and numbers on the left of the gel pictures indicate the predicted up or down-regulated in fruiting body and the value of log2-ratio. “-”represented the ESTs with no significant differences between the two samples by calculating the RPKM value. The right of the pictures shows the EST ID of the transcriptome and the cycles of PCR reaction. *Actin* (comp28675_c0) and *GAPDH* (comp26162_c0) primers were used to standardize RNA samples for each RT-PCR. Every pair of primers performed independent PCR reaction three times.

The mycelium and the fruiting body are two main developmental stages of mushrooms. To identify differentially expressed genes (DEGs) between the two developmental stages of *A. polytricha*, clean reads from dikaryotic mycelium and mature fruiting body were mapped back onto the ESTs respectively. The expression levels for each EST were calculated using the RPKM method [27]. A total of 12,834 (35.18%) ESTs were expressed in low abundance at both developmental stages. Out of 23,649 ESTs, 2,057 ESTs were significantly differentially expressed between the mycelium and fruiting body (FDR ≤0.001, |log_2_-ratio| ≥2), including 1,020 ESTs down-regulated and 1,037 up-regulated in the fruiting body (Figure 4A). Interestingly, 53 ESTs out of 2,057 DEGs were specifically expressed in mycelium and 377 ESTs in fruiting body. The absolute value of the log_2_-ratio ranged from 2.75 to 14.32 (Table S7).

#### 1) Validation of the DEG results by RT-PCR analysis

To validate expression profiles obtained by RNA-seq analysis, semi-quantitative RT-PCR were conducted to confirm the expression levels of 8 selected ESTs, with comp28675_c0 (*actin*) and comp26162_c0 (*GAPDH*) serving as the reference genes (Figure 4B, Table 3). All of these ESTs were amplified successfully and resulted into a single band. Four ESTs exhibited higher expression level in mycelium, including comp29169_c0, comp29186_c0, comp28312_c0 and comp29269_c0, while the other three were up-regulated in fruiting body, including comp27863_c1, comp10899_c0 and comp27971_c0. Comp21959_c0 exhibited same expression level in mycelium and fruiting body (Figure 4B). The expression patterns of these ESTs were consistent with the reads abundance of Illumina sequencing, suggesting that the DEG analysis was reliable.

**Table 3:** ESTs used for validation of gene expression profile in *A. polytricha* transcriptome data.

EST ID	Length (bp)	M_RPKM	F_RPKM	Log2(F_RPKM/M_RPKM)	Gene Description	Accession NO. of homolog (Organism)	E-value
comp28675_c0	1003	666.18	868.78	0.06	actin 1	EJD35981 (*Auricularia delicata*)	9.95E-169
comp26162_c0	833	477.24	385.23	−0.63	glyceraldehyde 3-phosphate dehydrogenase	EJD44578 (*Auricularia delicata*)	2.46E-117
comp29169_c0	2095	80.58	2.54	−5.18*	laccase	EJD39991 (*Auricularia delicata*)	0
comp27863_c1	461	4.90	75.82	5.52*	laccase	AAT73204 (*Auricularia polytricha*)	9.73E-77
comp29186_c0	949	110.52	7.03	−4.28*	PLC-like phosphodiesterase	EJD48564 (*Auricularia delicata*)	0
comp10899_c0	793	0.34	70.78	7.24*	hypothetical protein	EJD33907 (*Auricularia delicata*)	3.94E-116
comp29269_c0	1969	525.50	42.61	−3.94*	hydroxymethylglutaryl-CoA synthase	EJD47934 (*Auricularia delicata*)	0
comp27971_c0	985	11.18	244.27	4.13*	Aldo/keto reductase	EJD53165 (*Auricularia delicata*)	0
comp28312_c0	1096	320.67	10.42	−5.26*	hypothetical protein	EJD41350 (*Auricularia delicata*)	4.63E-70
comp21959_c0	741	36.31	75.24	0.73	hypothetical protein	EJD41368 (*Auricularia delicata*)	4.96E-12

*indicates a significant difference between mycelium (M_RPKM) and fruiting body (F_RPKM) (FDR≤0.001).

#### 2) Expression profiles of homologous TFs related to mushroom development

**Figure 5:**
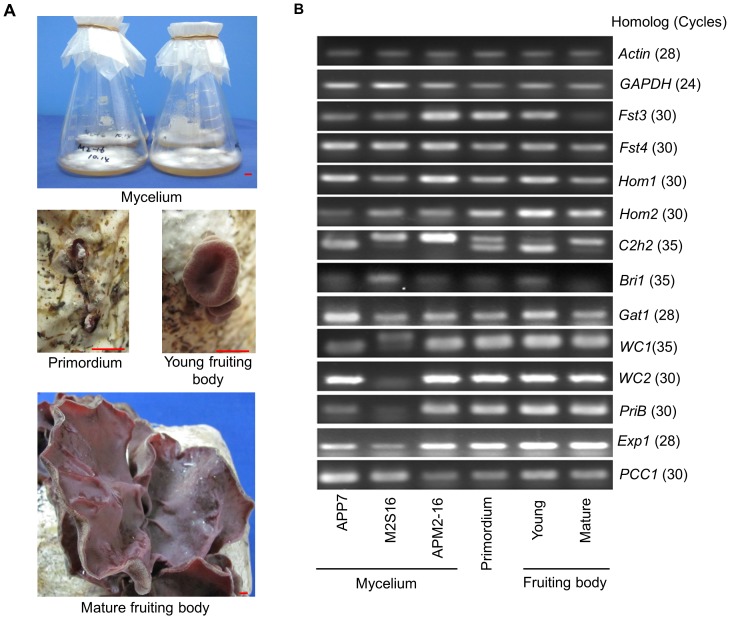
Expression profiles of homologous TFs in the development of *A. polytricha* fruiting body formation. (A) Different developmental stages in the life cycle of *A. polytricha* for samples collection of gene expression profiling; scale bars = 5 mm. The monokaryotic and dikaryotic vegetative mycelium were cultured in liquid CYM medium. The primordia were induced 5–7 d after wound treatment. The young fruiting bodies were cup-like and 3–5 mm in diameter. The mature fruiting bodies with spores were ear-like and more than 100 mm in longitudinal diameter. (B) Expression patterns of 12 homologous TFs estimated by semi-quantitative RT-PCR. The captions and numbers in brackets indicate the names of homologs and the cycles of PCR reaction, respectively. *Actin* and *GAPDH* were used to standardize RNA samples for each RT-PCR. At least three rounds of independent replications were used for each gene.

The expression profiles of ESTs encoding putative TFs were also investigated. Out of 1,349 putative TF encoding ESTs, 78 (5.78%) DEGs were detected when comparing the expression patterns of the mycelium and fruiting body, including 44 down-regulated and 34 up-regulated ESTs in the fruiting body (Table S6). Most differentially expressed ESTs between mycelium and fruiting body were classified into homeobox, C2H2 zinc finger and Zn2Cys6. Notably, all of the DEGs classified into homeobox were up-regulated in fruiting body. Whereas number of down-regulated ESTs encoding conserved protein domain Zn2Cys6 was more than four times that of up-regulated ESTs in fruiting body (Table S5). The homeobox and Zn2Cys6 transcription families might play important roles in the development of the fruiting body. TFs have been identified in some mushrooms, such as *A. bisporus*, *C. cinerea* and *S. commune* [8], [9], [35]. In *S. commune*, numerous TFs were found to be differentially expressed between developmental stages, suggesting that TFs were important developmental controls [8]. A set of TF genes, including *c2h2*, *fst3*, *fst4*, *hom1*, *hom2*, *gat1* and *bri1*, that are up-regulated during the formation of the primordia and/or mature mushrooms were inactive, demonstrating their important roles in the mushroom development of *S. commune* downstream of the mating-type loci [8], [18]. These TF genes have orthologs in *A. polytricha* transcriptome (Table 4). According to the DEG analysis of transcriptome, the ESTs that were homologous with *fst3* of *S. commune*, were significantly down-regulated in the fruiting body and the ESTs homologous with *c2h2*, *fst4*, *hom1*, *hom2*, *gat1* and *bri1* in the mycelium were similar to those of the fruiting body (Table 4). The homologous *A. polytricha* ESTs of these seven TFs were selected for semi-quantitative RT-PCR to estimate the gene expression profiles in different developmental stages, including the mycelium of two monokaryotic parents App7, M2S16, and the mycelium, primordium, young fruiting body and mature fruiting body of dikaryon hybridization APM2-16 (Figure 5, Table 4). Except the homolog of *c2h2*, all of the ESTs were amplified successfully and resulted into same size of bands between two monokaryotic parents. The expression levels of *fst3* and *hom1* homologs were the highest in the dikaryon mycelium and decreased gradually during the developmental of the fruiting body. The homolog of *fst4* exhibited similar expression levels between mycelium, primordium and fruiting body. The homologous ESTs of *hom2* and *gat1* showed higher expression level in the young fruiting body than that in dikaryotic mycelium. That two different size bands were amplified in App7 and M2S16 using primers for homologous EST of *c2h2* might represent a pair of allelic genes. The expression profiles of the candidate allelic genes were different in dikaryotic mycelium, primordioum, young and adult fruiting body. The expression level of *bri1* was lower than that of the other six genes and barely expressed in mature fruiting body. The expression profiles of these seven genes in dikaryotic mycelium and fruiting body, were conformed to the transcriptome data. However, the expression profiles of these genes were different from those in *S. commune*, *A. bisporus* and *L. bicolor* [18], [35]. This difference suggests that the developmental switches for fruiting body formation in *A. polytricha* and other agarics involve two distinct processes that occur after two monokaryons fuse to form dikaryons. WC-1 and WC-2, two proteins that form a photoreceptor and TF complex (WCC) and were cloned from the ascomycete *Neurospora crassa*, bind the promoters of light-regulated genes to activate transcription [36], [37]. The *dst1* and *phrA* genes are homologous genes of WC-1, cloned from *C. cinerea* and *L. edodes*, respectively [38], [39]. Furthermore, it has been shown in *L. edodes* that the WC-1 homolog PHRA interacts with the WC-2 homolog PHRB *in vitro* [40]. Searches of the genome databases suggest that *S. commune* and *L. bicolor* each have WC-1 and WC-2 homologs [41]. The expression of *WC-1* and *WC-2* homologous ESTs in dikaryotic mycelium and mature fruiting body of *A. polytricha* was similar (Table 4). The results from semi-quantitative RT-PCR showed that no significant differences of the expression level of *WC-1* and *WC-2* were existed in whole developmental processes of fruiting body, except that in the mycelium of M2S16 which may be caused by the incompatible primers for this parent (Figure 5B). The expression profile of *WC-1* homolog EST was consistent with the expression pattern of *phrA* but not consistent with *dst1* [38], [40]. However, the expression profile of *WC-2* homolog EST in *A. polytricha* was different slightly to that of *phrB* of *L. edodes* [40]. The deduced PRIB protein of *L. edodes*, a TF contains a Zn2Cys6 motif and a bZIP motif, was found to be most abundantly transcribed in the primordium and in the early stages of fruiting body formation [42]. In the *A. polytricha* transcriptome, one homologous EST for *PriB* was found. The result of semi-quantitative RT-PCR showed that the expression level of *A. polytricha PriB* in monokaryon was lower than that in different developmental stages of dikaryon (Figure 5B). The *exp1* transcription factor is involved in cap expansion and autolysis in *C. cinerea* [43]. The expression level of homologous *exp1* gene of *A. polytricha* was higher slightly in young and adult fruiting body than in mycelium and primordium (Figure 5B). Although the appearance of fruiting bodies for *A. polytricha* was different to the umbrella-like fruiting body for *C. cinerea*. the homologous *exp1* gene of *A. polytricha* may also play an important role during the ear-like fruiting body expansion. Another HMG protein, *pcc1*, plays an important role in promoting clamp cell development during the *C. cinerea A*-regulated pathway [44]. The homologous *pcc1* gene of *A. bisporus* was more highly expressed in the mushroom than in the mycelium and was down-regulated in the fruiting body of *L. bicolor* [35]. That the expression levels of the *pcc1* ortholog in different development stages of *A. polytricha* were similar indicated that the gene function of *pcc1* in different species was different. (Figure 5B, Table 4). Briefly, no significantly different expression level were existed in the dikaryotic mycelium and fruiting body except *fst3*, and the expression profiles of this homologous TFs in the course of *A. polytricha* fruiting-body formation was not completely same to other mushrooms. These results indicated that the formation of ear-like fruiting body of *A. polytricha* was different to other agaricomycetous mushrooms. However, the phenotypes resulting from the knockdown or knockout of these TFs should be determined to explore their actual functions in *A. polytricha* mushroom development.

**Table 4:** Expression of *A. polytricha* homologs of transcription factors that have been shown to be involved in mushroom development.

TFs	Protein ID (Organism)	Homolog in *A. delicata*	E-value^a^	EST ID	E-value^b^	TF family	M_RPKM	F_RPKM	Log2(F_RPKM/M_RPKM)
*fst3*	XP_003031320 (*S. commune*)	EJD43973	3.00E-63	comp22511_c1	5.15E-17	Zn2Cys6	164.06	4.68	−5.44*
				comp25466_c0	8.67E-140		1873.25	100.07	−4.54*
				comp30049_c0	7.00E-38		891.87	33.43	−5.05*
*fst4*	XP_003034563 (*S. commune*)	EJD36700	4.00E-72	comp1121_c0	3.33E-47	Zn2Cys6	1.98	7.85	1.59
				comp18748_c0	4.83E-175		4.04	6.69	0.40
				comp25058_c0	1.55E-163		12.07	10.84	−0.47
*hom1*	XP_003030056 (*S. commune*)	EJD52587	3.00E-20	comp29364_c0	0	Homeobox	228.93	49.72	−2.52
*hom2*	XP_003029756 (*S. commune*)	EJD42657	1.00E-23	comp23276_c0	4.77E-36	Homeobox	37.65	34.57	−0.46
				comp29656_c0	3.18E-12		249.22	247.11	−0.31
				comp30957_c0	5.47E-37		54.76	35.53	−0.95
				comp30958_c0	8.33E-42		34.71	17.97	−1.28
				comp30959_c0	6.23E-92		53.28	51.24	−0.38
				comp30960_c0	2.16E-72		134.41	79.40	−1.08
*c2h2*	XP_003026630 (*S. commune*)	EJD42424	1.00E-15	comp9578_c0	4.36E-27	C2H2 zinc finger	0.00	0.00	NA
				comp21593_c0	3.60E-115		18.42	11.72	−1.09
				comp22059_c0	8.05E-07		12.08	10.23	−0.58
				comp31864_c0	3.41E-19		7.36	2.95	NA
				comp37544_c0	8.31E-41		0.00	3.03	NA
*bri1*	XP_003038897 (*S. commune*)	EJD54129	2.00E-94	comp16842_c0	2.11E-58	AT-rich	11.37	17.66	0.30
				comp24328_c0	4.65E-60		14.56	21.21	0.21
				comp27551_c0	1.57E-73		16.96	23.73	0.22
				comp28003_c0	0		34.14	46.74	0.25
*gat1*	XP_003036589 (*S. commune*)	EJD53677	3.00E-39	comp10531_c0	9.49E-66	GATA type	326.48	64.18	−2.67
				comp22025_c1	7.09E-140		1023.83	222.41	−2.52
*WC1*	XP_003028974 (*S. commune*)	EJD49967	3.00E-64	comp29357_c0	3.01E-158	GATA type	25.31	94.02	1.57
*WC2*	XP_003035694 (*S. commune*)	EJD53150	8.00E-42	comp30604_c0	0	GATA type	393.15	390.09	−0.32
*PriB*	XP_001833558 (*C. cinerea*)	EJD53417	4.00E-89	comp30326_c1	2.09E-108	Zn2Cys6	73.21	59.07	−0.61
*exp1*	XP_001837422 (*C. cinerea*)	EJD53938	9.00E-87	comp30280_c0	0	HMG	80.77	165.41	0.74
*PCC1*	EKV47301 (*A. bisporus*)	EJD52624	1.00E-20	comp21444_c0	6.32E-16	HMG	98.44	124.60	0.01
				comp25704_c0	2.09E-71		42.57	37.35	−0.51

a indicates the identity between reference protein and the homologous protein of *A. delicata*.

b indicates the identity between the *A. delicata* protein and the homologous ESTs of *A. polytricha*.

*indicates a significant difference between mycelium (M_RPKM) and fruiting body (F_RPKM) (FDR≤0.001); NA means the expression differences were not analyzed because of the low expression level of mycelium and fruiting body.

#### 3) Discovery of genes related to fruiting body development by enrichment analysis

To study the functions of DEGs, functional annotation was adopted for these identified genes. GO annotation of DEGs was extracted and subjected to GO functional enrichment analysis. No DEG was found in the clusters of cell junction, nucleoid, symplast, virion, virion part, metallochaperone activity, protein binding transcription factor, receptor, immune system process, locomotion, positive regulation of biological process and reproductive process (Figure 1). These results indicated that the functions of the ESTs sorted into the twelve above-listed groups might have nothing to do with the development of the fruiting body. The number of ESTs with GO annotations up-regulated in the fruiting body (347) was greater than the number of down-regulated ESTs (303) (Table 5). GO functional enrichment analysis showed that many DEGs involved in “heme binding”, “iron ion binding”, “electron carrier activity”, “oxidoreductase activity”, “oxidation-reduction process” and “electron transport” (Table 5). More ESTs up-regulated in the fruiting body were involved in “metallopeptidase activity”, “ammonium transmembrane transporter activity”, “serine-type peptidase activity”, “urease activity”, “endopeptidase activity”, “ammonium transmembrane transport”, “cell adhesion” and “urea catabolic process”. These findings suggest that the biosynthesis, metabolism and assembly of proteins were more active in fruiting body development. In contrast, the GO categories “phosphatidylinositol phospholipase C activity”, “hydrolase activity, acting on glycosyl bonds”, “oligopeptide transport” and “diacylglycerol metabolic process” were over-represented in the up-regulated ESTs of the mycelium (Table 5).

**Table 5:** GO enrichment analysis in DEGs.

GO-ID	Term	Category	NO. of DEGsin subgroup	NO. of ESTsin subgroup	P-Value	FDR
**Up-regulated in mycelium**						
Total			303	11513	–	–
GO:0020037	heme binding	Molecular Function	33	440	5.39E-08	1.03E-04
GO:0005506	iron ion binding	Molecular Function	29	391	4.60E-07	3.95E-04
GO:0009055	electron carrier activity	Molecular Function	26	376	6.70E-06	3.08E-03
GO:0016491	oxidoreductase activity	Molecular Function	61	1322	7.17E-06	3.08E-03
GO:0004435	phosphatidylinositol phospholipase C activity	Molecular Function	3	4	7.08E-05	1.66E-02
GO:0016798	hydrolase activity, acting on glycosyl bonds	Molecular Function	21	311	7.43E-05	1.66E-02
GO:0055114	oxidation-reduction process	Biological Process	66	1344	2.92E-07	3.66E-04
GO:0006118	electron transport	Biological Process	31	431	3.55E-07	3.66E-04
GO:0006857	oligopeptide transport	Biological Process	4	6	6.77E-06	3.08E-03
GO:0046339	diacylglycerol metabolic process	Biological Process	3	4	7.08E-05	1.66E-02
**Up-regulated in fruiting body**						
Total			347	11513	–	–
GO:0020037	heme binding	Molecular Function	53	440	2.15E-18	4.52E-15
GO:0009055	electron carrier activity	Molecular Function	47	376	5.09E-17	5.25E-14
GO:0005506	iron ion binding	Molecular Function	47	391	2.42E-16	2.08E-13
GO:0016491	oxidoreductase activity	Molecular Function	77	1322	6.14E-09	2.88E-06
GO:0008237	metallopeptidase activity	Molecular Function	17	101	7.55E-09	3.25E-06
GO:0008519	ammonium transmembrane transporter activity	Molecular Function	4	5	3.96E-06	1.21E-03
GO:0008236	serine-type peptidase activity	Molecular Function	20	220	1.09E-05	2.43E-03
GO:0050660	flavin adenine dinucleotide binding	Molecular Function	18	199	3.20E-05	6.35E-03
GO:0009039	urease activity	Molecular Function	3	5	2.59E-04	3.82E-02
GO:0004175	endopeptidase activity	Molecular Function	19	258	3.04E-04	4.35E-02
GO:0055114	oxidation-reduction process	Biological Process	101	1344	3.62E-19	1.86E-15
GO:0006118	electron transport	Biological Process	48	431	2.37E-15	1.74E-12
GO:0072488	ammonium transmembrane transport	Biological Process	4	5	3.96E-06	1.21E-03
GO:0007155	cell adhesion	Biological Process	6	16	4.45E-06	1.21E-03
GO:0043419	urea catabolic process	Biological Process	3	4	1.06E-04	1.95E-02

KEGG pathway analysis showed that more DEGs participated to amino acid metabolism were existed in the metabolism group. In the category of cellular processes, more DEGs were involved in transport and catabolism. Signal transduction and translation were two subgroups containing more DEGs belong to environmental information processing and genetic information processing (Table S4). KEGG enrichment analysis showed that the down-regulated ESTs in the fruiting body were enriched for in carbohydrate metabolism relating to “Glyoxylate and dicarboxylate metabolism”. Large numbers of up-regulated ESTs in the fruiting body were associated with “Glyoxylate and dicarboxylate metabolism”, “fructose and mannose metabolism” and “amino sugar and nucleotide sugar metabolism” under the category of carbohydrate metabolism (Table 6). The enzymes of “fructose and mannose metabolism” and “amino sugar and nucleotide sugar metabolism” participate in the biosynthesis of polysaccharides. The monosaccharide compositions of *A. auricula* polysaccharides were as follows: glucose (72%), mannose (8%), xylose (10%) and fucose (10%) [45]. Genes involved in different pathways might have stage-dependent expression patterns in mushrooms [14]. The polysaccharide components in the mycelium and the fruiting body were different in *A. polytricha*. Except the ESTs with “amino sugar and nucleotide sugar metabolism” and “fructose and mannose metabolism” were highly expressed in the fruiting body, more ESTs up-regulated in the fruiting body were involved in “tyrosine metabolism” and “tryptophan metabolism” (Table 6). These results were in close accordance with the GO enrichment analysis. It indicated that the ESTs participating in these biosynthetic and degradation metabolism processes were essential to fruiting body development and that the analysis of this transcriptome was accurate and reliable.

**Table 6:** Representative mycelium-preferential and fruiting body preferential KEGG pathways.

Pathway_ID	Term	NO. of DEGs in the pathway	NO. of ESTs in the pathway	P-Value	FDR
**Up-regulated in mycelium**					
Total		56	2597	–	–
ko00480	Glutathione metabolism	4	34	5.56E-03	2.09E-02
ko00900	Terpenoid backbone biosynthesis	3	21	9.59E-03	2.88E-02
ko00910	Nitrogen metabolism	3	23	1.24E-02	3.10E-02
ko04142	Lysosome	4	48	1.87E-02	3.98E-02
ko00630	Glyoxylate and dicarboxylate metabolism	3	28	2.12E-02	3.98E-02
**Up-regulated in fruiting body**					
Total		49	2597	–	–
ko00140	Steroid hormone biosynthesis	3	8	3.31E-04	1.82E-03
ko00910	Nitrogen metabolism	4	23	7.61E-04	2.79E-03
ko00350	Tyrosine metabolism	4	26	1.23E-03	3.39E-03
ko00380	Tryptophan metabolism	4	39	5.66E-03	1.25E-02
ko00051	Fructose and mannose metabolism	3	23	8.57E-03	1.57E-02
ko03010	Ribosome	8	165	1.07E-02	1.69E-02
ko00630	Glyoxylate and dicarboxylate metabolism	3	28	1.48E-02	2.04E-02
ko00520	Amino sugar and nucleotide sugar metabolism	4	61	2.65E-02	3.24E-02

**Figure 6:**
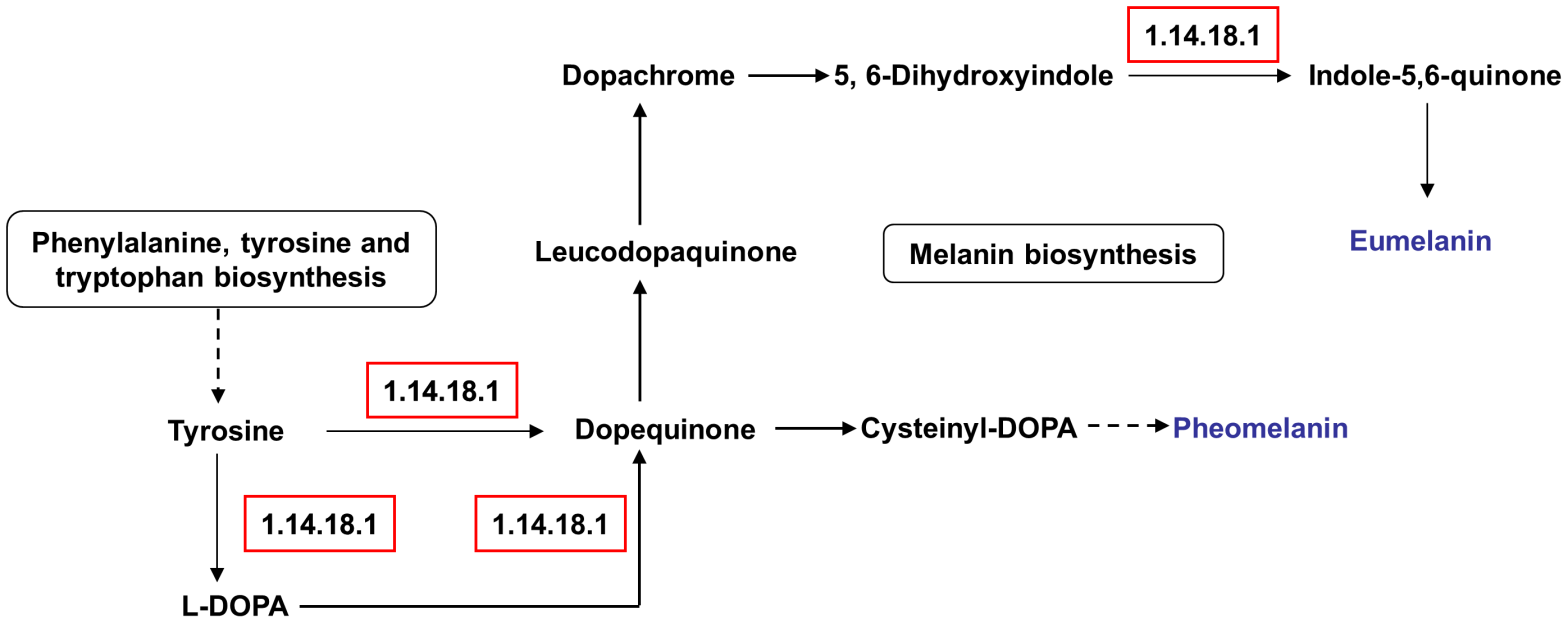
Putative components of melanin biosynthesis. Melanin biosynthesis is a part of tyrosine metabolism pathway. Red boxes represent genes up-regulated in fruiting body.

Amino acid metabolism, including the metabolism of tryptophan and tyrosine, was more active in the fruiting body (Table 6). Furthermore, the pathway of steroid hormone biosynthesis was highly expressed in the fruiting body, whereas terpenoid backbone biosynthesis was highly expressed in the mycelium (Table 6). This difference implies that different metabolites are synthesized in the mycelium and fruiting body of *A. polytricha*, and the nutrients might be different between these two developmental stages. Interestingly, the up-regulated ESTs in the fruiting body were enriched in the tyrosine metabolism, in which tyrosinase (EC 1.14.18.1) is involved in the biosynthesis of melanins (Figure 6). One out of two ESTs encoding tyrosinase (EC 1.14.18.1) were significantly up-regulated in the fruiting body. The expression levels of another EST were same in mycelium and fruiting body. Tyrosinase (EC 1.14.18.1), also often known as polyphenol oxidase (PPO) initiating an enzymatic brown reaction leading to the formation of brown or black pigments, is widely distributed in nature [46]. PPO from *A. bisporus* is liable to progress to enzymatic browning and pigmentation during development, post-harvest storage and pathogen infection [47], [48]. In *L. edodes*, the brown pigmentation of mycelial cells, one of the typical light-responses, is also thought to be attributable to the accumulation of melanin [40]. Thus, the black-brown color of the *A. polytricha* fruiting body could be related to the accumulation of melanins. The WC-2 homolog PHRB can bind to the promoter region of the *L. edodes* tyrosinase gene (*Le.tyr*) and these two genes are induced by light exposure, suggesting that PHRB can regulate the expression of the *Le.tyr* gene in a light-dependent manner [40]. Although the color of the mycelium will not become significantly darker under conditions of light compared to dark conditions, the formation and color of the *A. polytricha* fruiting body will be influenced by the light intensity. Therefore, it is worth further study to determine the functions of *Le.phrA*, *Le.phrB* and *Le.tyr* homologs during the different developmental stages of *A. polytricha*.

### Identification of SSRs

**Figure 7:**
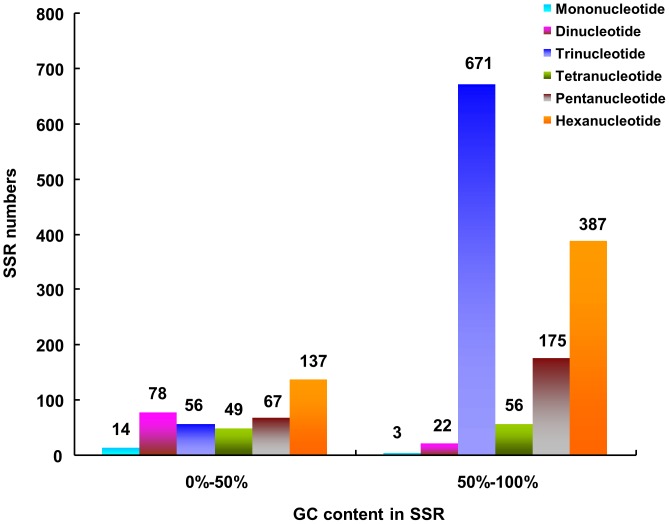
Distribution of the GC content in microsatellites among different nucleotide types found in the transcriptome of *A. polytricha*.

Transcript/EST-based molecular markers are an important resource for the construction of genetic maps, comparative genomics, genetic diversity analysis, functional genetic variation determination and molecular marker-assisted selection in breeding [49], [50]. For further assessment of the assembly quality as well as the development of molecular markers, all of the 36,483 ESTs generated in this study were used to mine potential microsatellites. Using the software SSR Locator, a total of 1,715 potential SSRs were detected in 1,502 (4.1%) ESTs of *A. polytricha*, of which 171 sequences contained more than one SSR, and 57 SSRs were present in compound formation (Table 7). On average, one SSR could be found every 11.3 kb in the transcriptome. The density of microsatellites in *A. polytricha* was similar to reports for other agarics, such as *A. delicata*, *C. cinerea*, *L. bicolor* and *G. lucidum* [51], [52]. Among the 1,715 potential SSRs, the tri-nucleotide SSRs represented the largest fraction (42.4%), followed by hexa-nucleotide (30.6%) and penta-nucleotide (14.1%) SSRs. Only a small fraction of tetra-nucleotide (6.1%), di-nucleotide (5.8%) and mono-nucleotide (1.0%) SSRs were identified in this transcriptome (Table 7). Over 50% of tri- and hexa-nucleotides were found in CDSs of the *G. lucidum* genome, whereas more than half of mono-, di-, tetra- and penta-nucleotides were distributed in intergenic regions [52]. Such a propensity for tri- and hexa-nucleotides in the coding regions may exist to suppress the other categories of SSRs, thus reducing the incidence of frameshift mutations in coding regions caused by nontriplet repeats [53]. The analysis of GC content in motif of microsatellites showed that most of the SSRs were rich in GC except mono- and di- nucleotide (Figure 7). In tri-nucleotide repeats, the microsaterllites with 50%–100% GC content were 10 times than that with 0–50% GC content. It was indicated that the GC content was more abundant in SSRs of the *A. polytricha* transcriptome. The identification of SSRs provides a very cost-effective option for the development of functional markers for various genetic studies.

**Table 7:** Statistics of SSRs identified in *A. polytricha* transcriptome.

Item	Numbers
Total number of sequences examined	36,483
Total size of examined sequences (bp)	19,384,088
Total number of identified SSRs	1,715
Number of SSR containing sequences	1,502 (4.1%)
Number of sequences containing more than one SSR	171
Number of SSRs present in compound formation	57
Frequency of SSRs (kb/one SSR)	11.3
Number of mono-nucleotide SSRs	17 (1.0%)
Number of di-nucleotide SSRs	100 (5.8%)
Number of tri-nucleotide SSRs	727 (42.4%)
Number of tetra-nucleotide SSRs	105 (6.1%)
Number of penta-nucleotide SSRs	242 (14.1%)
Number of hexa-nucleotide SSRs	524 (30.6%)

## Conclusions

To the best of our knowledge, this study is the first to analyze the difference between the mycelium and the fruiting body of *A. polytricha* by *de novo* transcriptome sequencing. Functional annotation and enrichment analysis of DEGs revealed that more ESTs up-regulated in the fruiting body stage were involved in tyrosine and tryptophan metabolism, fructose and mannose metabolism, amino sugar and nucleotide sugar metabolism, steroid hormone biosynthesis. Furthermore, many putative TF ESTs were identified by sequence comparison to known TF gene families of fungi. The expression profiles of some orthologs of TFs related to fruiting body formation in other agarics were investigated. The expression patterns of these TFs were different slightly to other mushrooms. Whether these genes play important role in the course of fruiting body formation should be further analyzed and verified, and extensive studies must be performed to understand the development of *A. polytricha*. However, numerous SSRs were predicted and can be used for subsequent marker development employed in strain typing, population genetics, phylogenetics, genetic linkage and QTL analysis. As such, the current study provides the largest number of transcripts to date and lays the initial groundwork for developmental and genetic research on *A. polytricha*.

## Supporting Information

Table S1 **Gene-specific primers for semi-quantitaive RT-PCR.**
(XLS)

Table S2 **Species distribution in Basidiomycota of transcriptome sequences with top BLASTx hits against nr database.**
(XLS)

Table S3 **11,513 ESTs from the** ***A. polytricha*** **transcriptome annotated by Gene Ontology (GO).**
(XLS)

Table S4 **Annotation of the** ***A. polytricha*** **transcriptome and differential expressed genes (DEGs) by KEGG classification.**
(XLS)

Table S5 **Number of homologous ESTs and DEGs in FTFD associated to different tanscription factor families.**
(XLS)

Table S6 **Predicted transcription factors of** ***A. polytricha*** **and their expression level (RPKM value) in vegetative mycelium and mature fruiting body.**
(XLS)

Table S7 **Differentially expressed genes (DEGs) between** ***A. polytricha*** **mycelium and fruiting body.**
(XLS)
